# Artificial Intelligence and Large Language Models in Surgical Shared Decision-Making and Informed Consent: A Narrative Review

**DOI:** 10.3390/healthcare14193331

**Published:** 2026-10-06

**Authors:** Nikoleta Koliou, Lazaros Kourtidis, Panagis M. Lykoudis, Ioannis Karavolias, Stavriana Charalampous, Georgios D. Ayiomamitis, Pinelopi Kouki, Christos A. Iordanou

**Affiliations:** 1 1st Surgical Department-Laparoscopic Unit, General Hospital of Piraeus Tzaneio, 18536 Piraeus, Greece; nicolekolioujd@gmail.com (N.K.); ioanniskaravolias@gmail.com (I.K.); stavrianachar@gmail.com (S.C.); agiogeo@gmail.com (G.D.A.); chriordanou@yahoo.gr (C.A.I.); 2 4th Department of Surgery, School of Medicine, National and Kapodistrian University of Athens, 10679 Athens, Greece; panlykoudis@med.uoa.gr; 3 2nd Department of Anaesthesiology, Attikon University Hospital, National and Kapodistrian University of Athens, 12462 Athens, Greece; penkouki@gmail.com

**Keywords:** artificial intelligence, large language models, informed consent, shared decision-making, patient decision aids, surgery, personalized risk, clinical governance

## Abstract

**Background:** Artificial intelligence (AI) and large language models (LLMs) may make surgical information easier to access and understand. However, these tools cannot replace the clinician’s role in shared decision-making or the requirements for valid informed consent. **Methods:** This narrative review follows the surgical patient pathway from diagnosis and explanation to option comparison, shared decision-making, and informed consent. It examines conventional digital tools, LLM-assisted communication, personalized prediction, direct consent studies, patient and professional perspectives and legal, ethical, and governance issues. A formal meta-analysis and study-level risk-of-bias assessment were not performed. **Results:** AI may support several stages of surgical care. Diagnostic and predictive systems can assist clinicians. LLMs can help explain approved information, prepare patients for consultation, and improve consent materials. Conventional interactive tools have the strongest evidence for better comprehension. Evidence for LLM-assisted consent is still based mainly on simulations and document analyses. Patient-facing use requires source control, privacy protection, and visible clinical oversight. AI cannot establish capacity, voluntariness, or final authorization. We propose a five-level framework in which safeguards increase with clinical influence. **Conclusions:** AI may support the patient’s path from diagnosis to an informed surgical choice when it uses approved information, serves a defined task, and remains under clinical oversight. Current evidence does not support autonomous AI-mediated consent or AI-directed treatment choice. The aim should be a better-informed, preference-sensitive decision, not an automated signature.

## 1. Background

Patient-centered surgical care requires more than accurate disclosure. Patients must understand the diagnosis, the proposed procedure, and reasonable alternatives. They also need to understand the expected benefits, possible harm, and important uncertainty. This information must then be considered in relation to their own goals and preferences [1]. Patient decision aids can support this process. They present options and consequences in a structured way, but they do not replace discussion with the clinical team [1]. Conventional digital tools provide an established basis for this role. Across systematic reviews, interactive interventions improved comprehension, knowledge, preparedness, decisional conflict, and decision quality [2,3,4]. These tools were most useful when patients actively reviewed information or explained it back. Their purpose is better deliberation, not greater uptake of surgery [1,2,3,4].

Artificial intelligence (AI) extends digital support to other parts of surgical care. Applications include clinical assessment, outcome prediction, intraoperative assistance, and workflow support [5,6,7,8,9,10,11,12]. However, specialty reviews show a gap between technical performance and prospective evidence of patient benefit [13,14,15,16]. Communication is therefore a central issue. Algorithmic output, clinical judgment, uncertainty, and patient values must be combined in a form the patient can understand [10,17].

Large language models (LLMs) are generative systems that produce language from patterns learned from large collections of text. They can translate specialist terms into plain language and reorganize dense material. They can also present approved institutional information in a conversational or multilingual format [18,19]. These functions may support education, documentation, and preparation before surgical consultation. Output is more reliable when the model uses a controlled knowledge base and a clearly defined task [18,19]. Performance still depends on the model, prompt, source material, clinical setting, and level of oversight. It can change between software releases, and as such, the model version must be recorded and reassessed after important updates [18,19,20].

LLMs must be distinguished from clinical prediction models. A validated prediction model estimates risk in a defined population. An LLM may help explain that estimate, but it should not generate an individual probability from a plausible-sounding answer. Keeping prediction and explanation separate reduces the chance that fluent language will be mistaken for calibrated evidence. It also preserves the distinction between prognosis and treatment recommendation [17,18,19,20,21,22,23,24,25,26].

LLMs also differ from fixed decision aids because their answers are generated dynamically. The same system may give a different answer when a question is repeated or rephrased. Possible failures include factual error, omission, inconsistent framing, and false reassurance [17,18,19,20]. Clinicians therefore need information about the system’s sources, validation, limitations, and common failure modes. Patients need a clear explanation of what the tool does and who remains responsible [17,18,19].

The aim of this narrative review is to distinguish the communication and decision-support roles of AI along the surgical patient pathway. It also proposes function-specific safeguards for integration into shared decision-making and informed consent.

## 2. Review Methodology

Two authors independently reviewed the literature using PubMed and Scopus. Searches were conducted from 1 June to 31 July 2026 and covered publications from 1 January 2015 to 31 July 2026. Three AI terms were used: “artificial intelligence”, “large language models”, and “ChatGPT”. Five clinical terms were used: “surgery”, “informed consent”, “patient communication”, “shared decision-making”, and “decision support”. Each AI term was combined with each clinical term using AND, giving 15 search pairs in each database. Appendix A lists all 15 pairs used in both databases. Their default search handling may differ.

The searches retrieved 2074 records. After deduplication, 953 records were screened by title. A total of 94 publications underwent full-text assessment and 53 were retained for the original narrative synthesis. Relevant publications addressed surgical communication, patient education, informed consent, prediction, or clinical decision-making across surgical specialties. Publications without clear relevance to surgical care or patient communication were excluded. The evidence included reviews, document analyses, feasibility studies, surveys, legal and ethical analyses, and prediction studies. The two reviewers extracted and discussed the evidence collaboratively. Disagreements were resolved through discussion and consultation with senior reviewers. Findings were organized by stage of the patient pathway and by the AI function examined. No meta-analysis or standardized risk-of-bias assessment was performed.

During revision, the presentation was guided by the six SANRA quality-appraisal domains and the publicly described VANRA principles [27,28]. SANRA is an appraisal tool rather than a reporting guideline. VANRA was listed by EQUATOR as a reporting guideline under development when this revision was prepared. We clarified the review’s aims and search process, distinguished study designs and clinical endpoints, and checked reference identity and bibliographic details against publisher or indexing records. Available primary texts were consulted for the principal quantitative claims and newly added technical examples. This does not establish full-text verification of every statement or formal checklist compliance.

## 3. From Diagnosis to Surgical Consent: A Patient-Centered Pathway

AI can support different tasks as the patient moves through surgical care. These tasks should remain distinct. A tool that helps identify a diagnosis is not the same as one that explains it, estimates prognosis, supports treatment choice, or prepares consent [1,2,3,4,17,18,19,21,22,23,24,25,26].

### 3.1. Diagnosis and Initial Clinical Assessment

AI is used in surgical imaging, lesion detection and classification, diagnostic support, outcome prediction, intraoperative guidance, and workflow management [5,6,7,8,9,10,11,12]. Specialty reviews describe applications in gastric, colorectal, hepatopancreatobiliary, and cancer care [13,14,15,16]. Most studies report technical performance. Few show that the system improves prospective patient care [13,14,15,16]. AI may therefore support the clinician’s assessment, but it should not be presented as an independent diagnosis [10,17].

LLMs have also improved clinician performance in structured diagnostic and medication-safety tasks when the task and evidence source were clearly defined [29,30]. These findings support supervised clinical decision support. They do not establish the safety of an open chatbot for diagnosis or surgical consent [29,30].

### 3.2. Explaining the Diagnosis and Care Pathway

Once the diagnosis has been established, the patient needs a clear explanation of what it means and what happens next. LLMs can translate specialist terms, shorten dense text, and present approved information in layers or in a conversational format [18,19]. They may help patients review information at their own pace and prepare questions. The output is safer when it comes from a controlled knowledge base and is reviewed by a clinician [17,18,19].

Model performance depends on the software version and clinical setting, as well as the prompt and source material [18,19,20]. Prompt design should therefore define a limited task rather than invite an unrestricted clinical answer. Instruction-based prompts can specify the audience, reading level, permitted source documents, and required output sections. For consent material editing, the instruction should preserve all material risks and alternatives, retain supplied numerical estimates, and identify missing information rather than invent it. Few-shot prompting adds clinician-approved examples of acceptable explanations. These examples should model neutral wording and preservation of clinical meaning rather than persuasion [31,32].

Chain-of-thought prompting uses examples or instructions that elicit intermediate reasoning steps. Its original evidence concerned arithmetic and other reasoning benchmarks [33]. Medical question–answering research has also tested a few examples, chain-of-thought, and self-consistency voting. Gains varied by benchmark; adding chain-of-thought did not always improve accuracy [32]. These results should not be interpreted as validation for patient-facing surgical consent. A persuasive reasoning trace is not evidence that an answer is correct. For clinical explanations, a brief rationale tied to approved sources is more useful for review than an unrestricted reasoning narrative.

An illustrative instruction for clinician-supervised editing is: “Use only the supplied approved leaflet. Rewrite it in plain language without removing material risks, benefits, or alternatives. Preserve every supplied number and time horizon. If information is missing, state this and refer the question to the surgical team. Organize the answer under procedure, benefits, risks, alternatives, and questions for the clinician.” This is a practical example, not a validated prompt. A grounded chatbot should additionally restrict retrieval to its approved source set and escalate unsupported questions. Clinicians should check omissions and factual consistency before release. Neither prompt wording nor retrieval guarantees safety [17,18,19,20,31,32].

AI can also create easy-language versions, draft translations, audio narration, captions, high-contrast text, and material that works with screen readers [18,19,34]. These features may improve access. However, machine translation is not a certified medical translation. A clinician should verify all material information, and professional interpreters or translators remain necessary when required [34,35].

### 3.3. Preparing for the Surgical Consultation

Conventional digital consent and decision-support tools have the strongest evidence. An updated systematic review reported improved comprehension with 11 of 13 interactive digital interventions. All three verbal discussion interventions using test/feedback or teach-back improved comprehension [2]. A surgery-specific review found better early comprehension in 21 of 30 studies [3]. Across 58 trials and 14,981 elective surgical candidates, decision-support interventions improved knowledge, preparedness, decisional conflict, and decision quality [4]. Effects on delayed recall, satisfaction, anxiety, and workflow were less consistent [2,3,4].

Before the consultation, a digital tool can explain the usual care pathway, summarize available options, and suggest questions. It can also ask which outcomes matter most to the patient [1,2,3,4,18,19]. This may help the surgeon focus on benefits, harms, alternatives, and uncertainty. No treatment or delayed treatment should be included when clinically reasonable, and surgery should not be presented as the default [1,2,3,4].

AI cannot correct every weakness in the consent pathway. Patients and clinicians still report limited consultation time, jargon, poor explanations, weak documentation, and poorly designed forms [36]. Digital support cannot replace adequate staffing, clinician training, or professional interpretation [2,3,4,36].

### 3.4. Discussing Options and Personalized Surgical Risk

After the diagnosis is understood, the patient and surgeon compare reasonable treatments. AI and machine learning models can combine clinical data, laboratory results, imaging, comorbidity, frailty, and procedure-specific factors to estimate outcomes [21,22,23,24]. Reported targets include mortality, complications, readmission, length of stay, stoma formation, recurrence, and function [21,22,23,24]. In 111,888 operations, models using preoperative and intraoperative data showed strong discrimination for several complications and generated patient-specific explanations [21]. Prospective evidence that these estimates improve decisions remains limited [22,23,24].

Colorectal surgery illustrates the limitations of prediction. Models have been developed for anastomotic leaks, infection, low anterior resection syndrome, permanent stoma, readmission, and mortality [23,24]. Performance may decline in other settings because patients and clinical practice differ. Outcomes are also affected by treatment choices, surveillance, and rescue. A prognostic estimate does not identify the best treatment or an individual treatment effect [21,22,23,24].

A model should be used only for a defined purpose, population, outcome, and time horizon. It requires internal and external validation, calibration, subgroup and fairness assessment, usability testing, and prospective evaluation [21,22,23,24,25,26]. Reporting should follow the Transparent Reporting of a multivariable prediction model for Individual Prognosis Or Diagnosis and Artificial Intelligence (TRIPOD+AI) statement [25]. The Prediction model Risk Of Bias ASsessment Tool plus Artificial Intelligence (PROBAST+AI) supports assessment of bias and applicability [26]. Institutions should monitor changes in case mix, clinical practice, data capture, and software. They should define when recalibration or suspension is needed [21,22,23,24,25,26].

When an LLM communicates a model-derived estimate, it should preserve the supplied probability and time horizon. It should not substitute an invented number or an unverified treatment recommendation [17,21,22,23,24,25,26].

### 3.5. Supporting Shared Decision-Making

Shared decision-making connects clinical evidence with what matters to the patient. The surgeon should first confirm that a risk model applies to the individual. Results should be presented as absolute frequencies over a defined period, with uncertainty stated clearly [1,4,21,22,23,24,25,26]. The same outcomes should be used when options are compared. The patient can then identify priorities such as survival, recovery, function, organ preservation, stoma avoidance, or quality of life. The record should include the estimate, its limits, the alternatives discussed, and the reason for the shared decision [1,4,21,22,23,24,25,26,37,38,39,40].

AI can support this discussion by organizing options, prompting questions, and summarizing the preferences stated by the patient. It should remain neutral. It should not infer values from demographic data, hide value judgments in option rankings, or use persuasive design to favor surgery or an institutional preference [1,4,17,37,38,41]. Communication-level personalization can adapt to language, detail, or format. Clinical personalization uses health data to estimate outcomes and therefore requires stronger validation and governance [18,19,21,22,23,24,25,26,39,40].

### 3.6. Supporting Informed Consent

Direct evidence remains limited to simulations and document analyses. In one study, ChatGPT-3.5, based on a generative pre-trained transformer (GPT), produced more complete and accurate descriptions than surgeon documentation for six procedures. Neither source reached the recommended reading level [42]. GPT-4 reduced the median grade level of forms from 15 academic medical centers from 13.9 to 8.9. Five newly generated and professionally reviewed forms reached a mean grade of 6.7 [31].

The Korean liver resection study provides an important caution. LLM editing shortened sentences and improved two readability measures, but reviewers rated risk descriptions and overall impression lower [35]. Simpler text can therefore weaken the perceived adequacy of disclosure. Readability alone cannot show that meaning has been preserved [2,3,31,35,42].

Studies in plastic, oral, and general surgery also reported shorter, more readable, or acceptable documents [43,44,45]. However, the text often remained difficult to read, important nuance was still needed, and agreement between reviewers was sometimes low. None of these studies showed better patient comprehension, decision quality, or clinical safety [31,35,42,43,44,45]. Representative direct evidence from these and related studies is summarized in Table 1.

These findings support clinician-reviewed preparation of consent material rather than its unreviewed release. The important question is whether simpler wording preserves material information and helps the patient deliberate [2,3,4,31,35,42,43,44,45].

### 3.7. Patient Experience and the Limits of AI-Mediated Consent

Patient-facing evidence is mixed. In a simulated tonsillectomy interaction, 86% found the information clear and 71% felt informed and satisfied, but only 42% were reassured about data security [46]. In a randomized United Kingdom (UK) vignette study, 87.6% judged AI-supported consent valid, yet satisfaction was 59.5%. Satisfaction exceeded 93% when the same exchange was attributed to a junior doctor or surgeon [47]. Clear information did not replace the relational value of human care [46,47,48].

Conversational tools can answer questions and prompt teach-back [2,3,18,19,46,47]. They may flag uncertainty or unanswered questions for clinical review. However, neither a fluent answer nor a completed teach-back prompt independently establishes decision-making capacity, voluntariness, or valid consent. Assessment must remain part of the clinician-led encounter [1,37,38,46,47,48].

Emergency surgery, impaired capacity, and pediatric care require additional safeguards. Time pressure does not remove the need to seek consent from a patient who can decide. Where capacity is absent, clinicians must identify the lawful authority for treatment and consider known wishes and the relevant representative or best-interests process. UK professional guidance illustrates that immediately necessary emergency treatment may proceed when a patient lacks capacity and their wishes cannot be established [49]. In pediatric care, age-appropriate involvement and assessment of the young person’s ability to decide are required alongside parental authority where applicable [50]. Assent and parental permission are not interchangeable with the child’s own valid consent. The governing rules differ between jurisdictions. AI may help explain information to patients or representatives, but it must not determine capacity or treatment authority or delay urgent care. Evidence from routine document editing and simulated adult encounters cannot be assumed to apply to these settings [31,35,42,43,44,45,46,47,51].

AI-assisted records may support continuity and investigation of unsafe output. Patients should know what is recorded and how it is used. Retention and access arrangements should be explained when relevant [18,19,37,38,39,40,41,52]. Level-specific documentation is discussed in Section 4.6.

## 4. Legal, Regulatory, and Governance Considerations

### 4.1. Consent, Disclosure, and Materiality

The phrase ‘consent for medical AI’ can refer to three different issues. The first is valid consent to the clinical intervention. The second is whether the role of AI in producing information, predictions, or recommendations is material to that decision. The third is lawful processing of personal and health data. These issues overlap, but they are not the same [37,38,39,40]. Legal analyses from the United States, United Kingdom, and Germany suggest that existing consent law can address AI through established concepts. These include material risk, reasonable alternatives, experimental use, and relevance to the individual patient. More detailed disclosure may be required during experimental or transitional use [37,38].

Disclosure should reflect the role and risk of the system. Routine grammar editing of clinician-approved text may not require individual disclosure, although institutions should be transparent about its use. An interactive chatbot, a patient-specific risk estimate, or an AI-ranked list of options is different and should be disclosed in plain language. Relevant questions include whether the output reaches the patient, uses identifiable data, changes the information or recommendation, introduces uncertain risk, or would matter to a reasonable patient. Validation, regulation, and the availability of a non-AI option are also relevant. Patients should be told what the system does, what its limits are, how clinicians review its output, and how to request another pathway [37,38].

Two errors are possible: AI exceptionalism treats every hidden software contribution as a separate consent issue and may distract from information that is genuinely material, or AI normalization treats generative or predictive systems as ordinary software even when their output is uncertain or influences treatment. A proportionate approach focuses on the system’s actual role in the care pathway. It asks how an error could affect understanding or choice, whether the error can be corrected before action, and which safeguards preserve meaningful clinical involvement [37,38].

### 4.2. Data Protection and Regulation

AI-assisted surgical decision support may process health data, which receive enhanced protection under the General Data Protection Regulation (GDPR). Under Article 6 and 9 of the GDPR, processing requires an appropriate lawful basis and a condition for special-category health data. Purpose limitation and data minimization apply alongside transparency and security. Articles 25 and 32 of the GDPR address data protection by design and by default and security measures appropriate to the risk, respectively. Article 22 provides specific protection concerning solely automated decisions with legal or similarly significant effects [53]. Consent is not a substitute for privacy by design, confidentiality, access control, or secure infrastructure [39,40,53]. Konda et al. describe a predictive framework that combines encryption and decryption with subsequent feature selection and neural network analysis [54]. This is a technical example of combining protected data handling with analytics, not evidence that a surgical consent application prevents breaches or meets GDPR requirements.

Practical privacy by design begins with mapping the data flow. Administrators should specify what reaches the model provider and whether prompts or outputs are retained or reused. Controls should include data minimization, role-based access, encryption in transit and at rest, managed cryptographic keys, and risk-appropriate retention. Pseudonymization can reduce exposure but is not the same as anonymization. Contracts and local security reviews should address provider access and secondary use. Encryption does not protect plaintext once an authorized service decrypts it for processing [39,40,41,53,54].

Federated learning offers a different architecture for collaborative model training. Hospitals keep source datasets locally and exchange model updates rather than pool raw patient records. The EXAM study used this approach across 20 institutions to predict clinical outcomes in COVID-19 [55]. This example concerns prediction-model development, not consent dialogue. Federated training still needs protected communications and governance of participating sites and updates. It also does not make a later inference service private if identifiable patient prompts are sent to an external provider [41,55].

Homomorphic encryption permits specified computations on encrypted data without the computing service first seeing the plaintext. CryptoNets demonstrated encrypted neural network prediction as proof of concept [56]. Its model and computational constraints differ from those of a general-purpose conversational LLM. Hospitals should assess whether encrypted computation is feasible for the intended model and response time rather than assume it can be added to any chatbot. Federated learning and homomorphic encryption address different parts of the data pathway. They complement access control and security governance; neither establishes lawful processing or GDPR compliance by itself [41,53,56].

Software intended for a medical purpose may also be regulated as a medical device. The European Union Artificial Intelligence Act (EU AI Act) provides a separate legal framework based on intended purpose and statutory classification [57]. Its requirements for relevant systems include risk management and data governance as well as documentation, logging, transparency, human oversight, and cybersecurity. The five operational levels proposed in this review do not replace those legal categories or a regulatory assessment. Conformity alone also does not establish suitability for a particular population or clinical workflow [37,38,39,40,41,52]. Institutions must check the current legislation and applicable national rules before deployment.

### 4.3. Responsibility and Documentation

AI does not take over the clinician’s duty to explain material risks, discuss reasonable alternatives, and respond to the patient’s circumstances and values. Clinicians and institutions remain responsible for the systems they select, configure, approve, and use [1,37,38]. Responsibility cannot be reduced to a general statement that a human is ‘in the loop.’ Patients should be able to identify the clinician and institution responsible for the information used in their care [37,38,39,40,41,52].

Documentation should be sufficient to explain how material AI output contributed to the consent process or treatment decision. When patient-specific dialogue or prediction is used, the record should identify the reviewed output, its main limitations, and the clinician’s interpretation [21,22,23,24,25,26,37,38,39,40,41,52]. This supports accountability without transferring responsibility to the system.

### 4.4. Development and Interpretation of the Five-Level Framework

The authors developed the framework through narrative synthesis of the reviewed literature and discussion of needs identified in surgical communication and decision-making. All authors contributed through their respective roles and approved the final framework. Five levels were selected to separate functional transitions with different consequences for information quality, patient autonomy, and clinical influence. This was a literature-informed author-consensus process, not a formal Delphi exercise or a statistical derivation.

The framework classifies AI by its function in the surgical pathway rather than by its technology label. The five levels distinguish backstage editing, static patient-facing information, interactive education, patient-specific prediction, and treatment influence. These transitions change how an error can affect understanding or choice. They also change whether an error can be corrected before it reaches a patient. The levels therefore organize operational safeguards; they are not a validated numerical risk score [17,25,26,37,38,41,52]. Five dimensions inform classification:

1. **Clinical influence:** Identify whether the system edits language, provides education, estimates outcomes, ranks options, or recommends treatment [17,37,38,41].
2. **Effect on autonomy:** Assess whether an error could distort understanding, omit a material alternative, pressure the patient, or compromise voluntariness [1,17,37,38,48].
3. **Boundaries of the output:** Determine whether the content is fixed and approved or generated dynamically in response to open questions [17,18,19].
4. **Personalization and data sensitivity:** Establish whether the system uses general information or identifiable health data [21,22,23,24,25,26,39,40].
5. **Evidence and reversibility:** Consider whether an error is likely to be found before the encounter or could affect a time-sensitive decision [25,26,41,52].

The highest-influence function in actual use determines the operational level. A chatbot that only explains approved general information is Level 3. If it communicates an individual estimate from a predictive model, that function requires Level 4 safeguards. If it ranks treatments, Level 5 applies. Validation and clinician review may reduce residual risk, but they do not downgrade a predictive or treatment-influencing function to a language-editing category [17,21,22,23,24,25,26,37,38,41,52].

**Figure 1:**
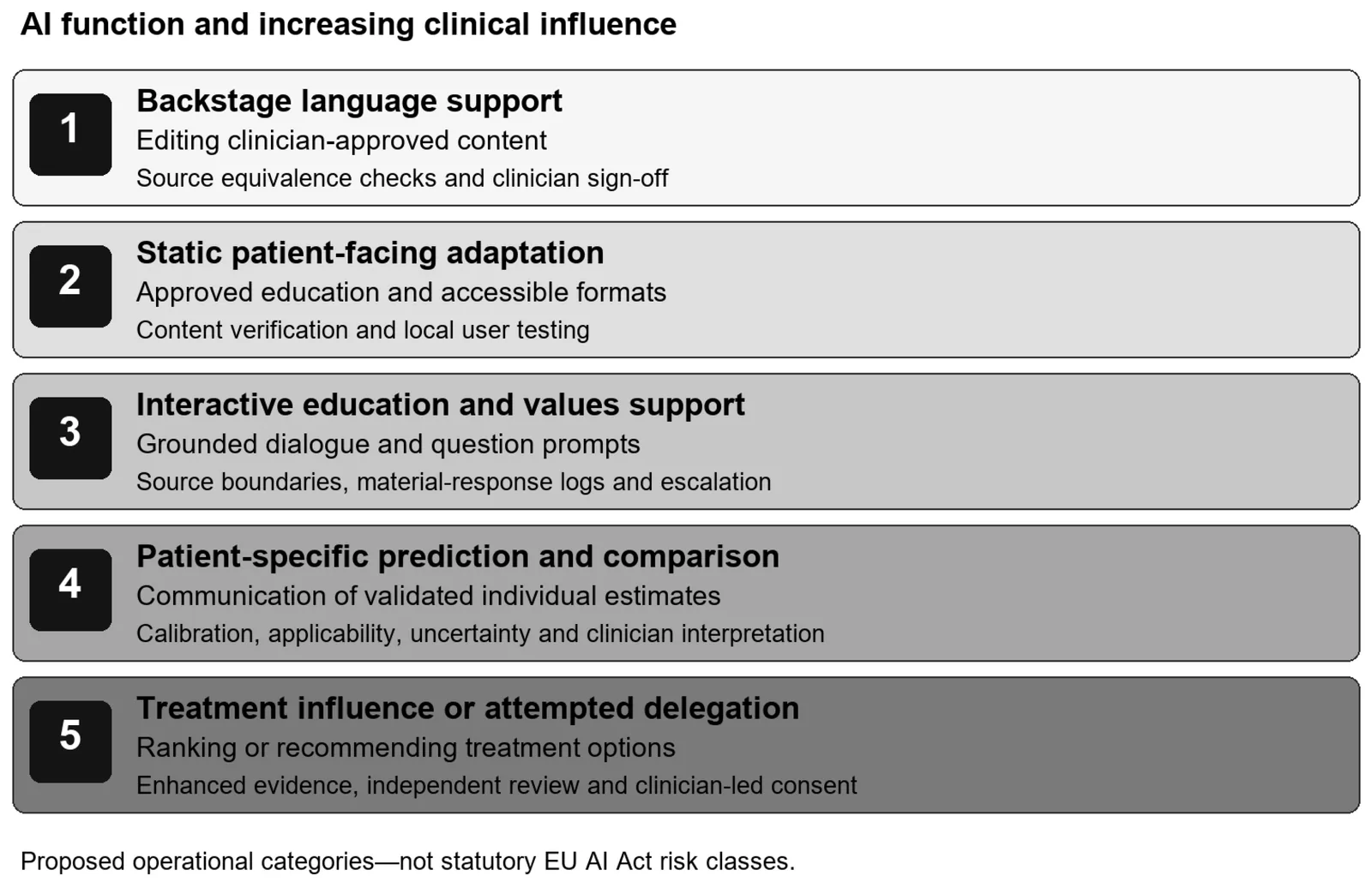
Proposed operational levels for AI-supported surgical communication and decision-making. Numbered labels and progressively darker gray-scale panels distinguish all five levels without reliance on color. The categories are conceptual and do not correspond to EU AI Act statutory risk classes [17,25,26,37,38,41,52,57].

The framework is complementary to, but distinct from, the EU AI Act, FUTURE-AI, and DECIDE-AI. The EU AI Act determines legal obligations through its statutory criteria [57]. FUTURE-AI provides lifecycle guidance for trustworthy health AI [41]. DECIDE-AI concerns reporting of early live clinical evaluation [52]. These sources informed the safeguards but did not supply or validate the five categories. Dr. Effie Simou provided informal expert feedback. The framework has not undergone formal independent validation or prospective testing. Its classification reliability and clinical utility remain questions for future research. Figure 1 summarizes the progression across the five levels. Table 2 presents the functions, examples, safeguards, and proposed deployment positions for each level.

### 4.5. Worked Vignette: Escalation as Functions Are Added

Consider an illustrative preoperative tool for a patient discussing colorectal surgery. Initially, it presents a clinician-approved leaflet in plain language. This is Level 2 because the patient receives static general information. Adding a chatbot that answers questions only from the approved leaflet moves the tool to Level 3. Dynamic responses now require source boundaries and escalation of unsupported questions. If the interface then communicates an individual complication estimate produced by a separately validated predictive model, it moves to Level 4. The estimate requires calibration and applicability checks with clinician interpretation. The LLM should only explain the supplied estimate. Adding a function that ranks reasonable treatment options moves the tool to Level 5 because the output can shape treatment choice. This requires stronger evidence and explicit review of the ranking’s assumptions and influence. The surgeon and patient retain responsibility for deliberation and the lawful consent process. The vignette demonstrates classification logic only; it does not represent a tested system or validated clinical pathway.

### 4.6. Cross-Cutting and Level-Specific Safeguards

Every level requires accountable governance and protection of any personal data processed. The depth of validation, logging, monitoring, and corrective action should then increase with clinical influence. The following safeguards combine this common baseline with function-specific requirements [17,25,26,37,38,41,52]:

1. **Governance and intended use:** Define the clinical decision, users, setting, and target population. State which outputs are permitted or prohibited and provide a non-AI pathway. Name accountable owners for clinical content, model validation, procurement, data protection, cybersecurity, incident response, and post-deployment monitoring [37,38,39,40,41,52].
2. **Evidence and validation:** Match testing to risk. Static editing requires content-equivalence checks, whereas predictive systems need external validation, calibration, subgroup analysis, and clinical impact assessment [21,22,23,24,25,26,41,52].
3. **Knowledge control and versioning:** Use approved sources and retrieval boundaries with task-specific prompts. Record changes to sources, prompts, and software. Re-test after material updates. Prompt controls support a bounded workflow but are not proof that hallucinations or omissions have been eliminated [17,20,32,41,52].
4. **Active human oversight:** Define which outputs are reviewed, who reviews them, when reviews occur, and how disagreement or escalation is handled. Oversight must be practical within the workflow [17,37,38,39,40,41,52].
5. **Patient transparency, comprehension, and choice:** Disclose AI when its role is material. Explain its limits and the extent of human review, support teach-back, preference, and clarification, and maintain access to clinician-led or non-AI care [1,2,17,37,38,39,40,41,52].
6. **Documentation, monitoring, and incident response:** At levels 1–2, retain the approved source and released version with equivalence checks and clinician sign-off. Correct or withdraw inaccurate materials and assess whether affected patients require updated information. At Level 3, record material responses and corrections with escalation events; recurrent unsupported answers should trigger restriction or suspension of the affected function. At Level 4, add the input provenance, predictive model version, estimate, uncertainty, and clinician interpretation. Monitor calibration, subgroup performance, and drift against predefined review or suspension thresholds. At Level 5, also document the basis for rankings and their influence on the decision, with independent clinical review of unsafe recommendations. Retain only the information necessary for clinical accountability and security. Privacy and cybersecurity incidents require an appropriate response at every level, including legally required notification where applicable. An output that could change treatment requires more urgent containment than a minor wording error, followed by investigation and risk-matched revalidation [21,22,23,24,25,26,37,38,39,40,41,52,53,57].

These safeguards reflect the FUTURE-AI principles of fairness, universality, traceability, usability, robustness, and explainability [41]. DECIDE-AI supports reporting of early live clinical evaluation [52]. Randomized trials of AI interventions should use the CONSORT-AI extension [58]. None of these guidelines validates the proposed five-level framework. Generative systems should not invent individual risks or use hidden value assumptions to favor a treatment [17,21,22,23,24,25,26,37,38,41].

### 4.7. Staged Implementation

Implementation should start with supervised functions of clearly defined values. Examples include plain-language editing of approved content and pre-consultation education [18,19,31,35,41,42,43,44,45,52]. Identifiable patient information should not be entered into unapproved consumer chatbots. Source-grounded retrieval and standardized task instructions are sensible controls, but their ability to reduce hallucinations and omissions in a specific consent pathway must be tested rather than assumed [17,18,19,20,32,39,40,41,52].

Deployment should be gradual. Offline testing should cover representative questions, uncommon but important risks, language variants, and foreseeable misuse. A limited pilot should follow before wider use. During the pilot, clinicians should observe how patients use the system, remain easy to contact, and review corrections and overrides. Predefined stopping rules are also needed [41,52]. Success should not be judged only by accuracy or completion rates. Evaluation should ask whether the system creates more time for deliberation, preserves trust, improves decisions, and avoids unequal access or additional documentation burden [2,3,4,41,46,47,51,52,59,60].

## 5. Discussion

### 5.1. Principal Findings and Clinical Implications

The main finding of this review is that AI cannot be treated as a single intervention in surgical consent. Evidence, risk, and governance depend on the function being used. Conventional interactive education has the strongest evidence base. Clinician-supervised LLM editing is promising but less established. Evidence for open conversational systems remains preliminary, and current data do not support autonomous consent or AI-directed treatment choices [2,3,4,17,21,22,23,24,25,26,31,35,37,38,41,42,43,44,45,46,47,52].

Consent document quality, decision-aid quality, and valid authorization are related but different outcomes. A complete document may still frame options unevenly or fail to support the patient’s values. A useful interactive tool may improve knowledge without establishing valid consent. AI is therefore best considered as support before and around the clinical encounter, with individual interpretation and the consent process remaining clinician-led [1,2,3,4,37,38,46,47,48].

The proposed five-level framework links these distinctions to operational safeguards. Low-influence tools require source control and content review. Interactive, predictive, and treatment-influencing functions require progressively stronger evidence and oversight. This is a literature-informed proposal for proportionate governance, not a means of avoiding regulation. Its reproducibility and clinical usefulness still require independent and prospective evaluation [17,21,22,23,24,25,26,37,38,39,40,41,52].

### 5.2. Trust, Equity, and Professional Readiness

Patient-facing studies show cautious interest alongside continuing concerns about privacy, empathy, and accountability. Trust depends on reliable performance, visible clinician involvement, clear disclosure of limitations, and a meaningful choice about how the interaction takes place. A system may use respectful language, but it should not imply that it understands or cares. Its role is to communicate appropriately and flag concerns that require human attention [46,47,48,61,62,63,64]. Ethical reviews across medicine, oncology, and surgery identify similar concerns about bias, explainability, accountability, professional responsibility, and preservation of human judgment [61,62,63,64].

AI may improve access through adaptable language and format. It may also widen disparities through unequal digital access, limited language coverage, unrepresentative data, or poorly calibrated estimates. Translation errors and poor calibration may place the greatest burden on patients who already face barriers to care. Equity should therefore be assessed using subgroup-specific error rates, comprehension, uptake, completion, and trust. Professional interpretation, accessible formats, and face-to-face support should remain available [21,22,23,24,25,26,34,35,39,40,61,62,63,64].

Professional readiness is a separate constraint. Governance should involve surgeons, nurses, patient representatives, interpreters, and health-literacy specialists. Ethicists, legal and data protection advisers, informaticians, and human factors experts should also be included [37,38,39,40,41,51,52,59,60,61,62,63,64]. Clinicians need practical AI literacy. This includes intended use, quality of evidence, uncertainty, bias, privacy, common failure modes, and responsibility for overriding the system. Both uncritical trust and reflexive rejection can compromise care. Oversight must be informed, active, and workable in routine practice [41,51,52,59,60].

## 6. Limitations

This narrative review is limited by its search coverage and heterogeneous evidence base. Only PubMed and Scopus were searched; therefore, relevant technical, legal, and interdisciplinary literature indexed elsewhere may have been missed. Title-based screening and default-field term pairs may also have omitted relevant publications. The search ended on 31 July 2026 and cannot represent all subsequent developments. Most LLM consent studies examined documents or simulated encounters rather than real surgical decisions. Changing commercial models also make comparisons unstable [20,31,35,42,43,44,45,46,47]. No meta-analysis or standardized study-level risk-of-bias assessment was undertaken. Reported improvements in readability or acceptability should not be interpreted as causal evidence of better consent or clinical safety [2,3,31,35,42,43,44,45,46,47]. The five-level framework has not undergone formal independent or prospective validation. Privacy and prompting examples drawn from other settings illustrate technical options but do not validate a surgical consent workflow. Legal applicability remains jurisdiction-specific [37,38,39,40].

## 7. Future Research

Research should move beyond readability scores and document quality ratings [2,3,4,31,35,42,43,44,45,46,47,65]. Priorities include the following:

1. Prospective pathway trials comparing usual care, conventional decision aids, static AI-assisted materials, and bounded interactive tools. Outcomes should include knowledge, risk comprehension, decisional conflict, preparedness, and value concordance. Trust, anxiety, consultation time, clinician workload, regret, and safety should also be measured [2,3,4,31,35,41,42,43,44,45,46,47,52].
2. Direct assessment of comprehension, including understanding of risks, benefits, alternatives, uncertainty, and the option of no treatment. Reviews of risk communication suggest possible benefits but also substantial heterogeneity and a risk of bias [2,3,4,65].
3. Multilingual and low-literacy co-designs that measure semantic accuracy and culturally relevant decision outcomes rather than language metrics alone [34,35].
4. Evaluation of validated personalized estimates presented in numerical, visual, narrative, and conversational formats. Studies should assess calibrated risk perceptions and effects on patient preferences [21,22,23,24,25,26].
5. Transparent reporting of model version, prompts, retrieval sources, and settings. Reports should also describe guardrails, clinical review, and error handling. Research should examine automation bias, overreliance, deskilling, workflow burden, and the quality of clinician override [17,20,25,26,41,52].
6. Long-term study of equity, governance, and liability. Surveillance should include subgroup performance, material disclosure, responsibility, and model drift. Near misses, complaints, and performance after updates should also be recorded [37,38,39,40,41,51,52,59,60,61,62,63,64].
7. Independent evaluation of the five-level framework, including agreement between raters classifying real systems and prospective assessments of whether level-matched safeguards identify errors and support clinical decisions. Emergency and pediatric settings should be examined separately before extrapolating findings from routine adult care.

## 8. Conclusions

Digital tools can support patient preparation and communication of surgical information. Evidence is strongest for conventional interactive education. LLM-assisted editing has encouraging document-level findings but requires clinical review [1,2,3,4,18,19,31,34,35,42,43,44,45,46,47]. Current evidence does not support autonomous consent or unverified patient-specific prediction. Clinical usefulness should be judged by comprehension and preference-sensitive decisions rather than readability alone [17,21,22,23,24,25,26,31,35,37,38,39,40,42,43,44,45,46,47].

The proposed five levels organize safeguards according to the function being used. They distinguish bounded communication support from prediction and treatment influence, while retaining clinician-led consent and accountability. Independent and prospective evaluation is needed before the framework can be considered validated [17,25,26,37,38,41,52].

## Figures and Tables

**Table 1:** Representative direct evidence on LLM-assisted surgical consent and communication [31,35,42,43,44,45,46,47].

Study	Design and Material	Main Findings	Interpretation
Decker et al., 2023 [42]	Cross-sectional; 36 descriptions for 6 procedures; ChatGPT-3.5 vs. 30 surgeon entries	Composite score 2.2 vs. 1.6 (*p* < 0.001); reading grade 12.9 vs. 15.7 (*p* = 0.10); neither met the sixth-grade target	Promising, but based on a small sample without patient participants
Ali et al., 2024 [31]	GPT-4 editing of forms from 15 academic centers; 5 de novo forms; medical and legal review	Median grade fell from 13.9 to 8.9; reviewed content retained legal and clinical sufficiency; de novo forms averaged a reading grade of 6.7	Supports multidisciplinary review; comprehension and clinical outcomes were not assessed
Patel et al., 2024 [43]	10 documents for 5 esthetic procedures; ChatGPT-4 vs. professional society forms; 5 surgeon reviewers	AI-generated forms were shorter (1023 vs. 2901 words) and easier to read (grade 11.2 vs. 12.5); major quality domains were similar	Both sources remained above the recommended reading level and complication descriptions still required nuance
Vaira et al., 2025 [44]	30 documents for 10 oral procedures; ChatGPT-4, Gemini Advanced, and resident author; 18 reviewers	ChatGPT-4 generally received the highest accuracy, completeness, and readability ratings; the documents remained difficult to read according to formal readability measures	Expert ratings favored ChatGPT-4, but the study did not assess patient outcomes
Oh et al., 2025 [35]	7 Korean liver resection forms edited by ChatGPT-4o; 7 institutions and 7 specialists	Readability improved significantly, but ratings of risk descriptions and overall impression declined	Simplification may weaken the perceived adequacy of risk disclosure
Helbing et al., 2026 [45]	Feasibility survey; 21 surgeons and residents; 4 common procedures	Mean agreement on acceptability ranged from 94.5% to 96.4%, with low interrater reliability and missing nuance identified	Supports supervised drafting, not release without clinical review
Teasdale et al., 2024 [46]	Simulated tonsillectomy dialogue; 86 lay participants and health care professionals	86% found information clear; 71% felt informed and satisfied; 42% were reassured about data security	Generally acceptable, but privacy and relational concerns remained
Allen et al., 2026 [47]	Randomized vignette study; 376 UK participants; identical transcript attributed to AI, junior doctor, or surgeon	Overall, 87.6% judged AI-supported consent valid, but satisfaction was 59.5% vs. >93% for the human conditions	Equivalent information did not produce equivalent satisfaction

Abbreviations: AI: artificial intelligence; GPT: generative pre-trained transformer; LLM: large language model; UK: United Kingdom; vs.: versus.

**Table 2:** Five-level framework for responsible AI integration in patient-centered surgical decision support. Deployment positions describe the proposed clinical governance approach, not a statutory classification [17,25,26,37,38,41,52,57].

Level	Function	Example	Required Safeguards	Deployment Position
1	Backstage language support	Grammar, formatting, and controlled plain-language editing	Approved source; equivalence check; named clinician approver; version record	Appropriate for supervised routine use
2	Static patient-facing adaptation	Layered education, accessibility, or translated draft	User testing; human verification; accessibility review; non-AI alternative; update cycle	Use after local approval; professional translation where material
3	Interactive education and values support	Grounded chatbot, question prompts, and teach-back cues	Explicit AI notice; bounded knowledge; safety triggers; logging; rapid clinician escalation; prospective evaluation	Pilot under active monitoring before scale-up
4	Patient-specific prediction and option comparison	Anastomotic leak, mortality, stoma, readmission, or functional outcome estimate	Regulatory assessment; external validation; calibration and fairness; uncertainty; clinician interpretation; predictive-model and input provenance; risk-matched audit trail	Use only within governed clinical pathways after model and regulatory assessment
5	Treatment influence or attempted consent delegation	Option ranking, treatment recommendation, capacity/voluntariness assessment, or final authorization	Enhanced evidence and disclosure; transparent ranking assumptions; independent clinical oversight; continuous decision influence; no autonomous capacity determination or treatment authorization	Clinician-led deliberation and lawful consent; autonomous consent delegation is not supported

Abbreviation: AI: artificial intelligence.

## Data Availability

No new data were created or analyzed in this study. Data sharing is not applicable to this article.

## Abbreviations

AI	Artificial intelligence
LLM	Large language model
TRIPOD+AI	Transparent Reporting of a multivariable prediction model for Individual Prognosis Or Diagnosis and Artificial Intelligence
PROBAST+AI	Prediction model Risk Of Bias ASsessment Tool plus Artificial Intelligence
GPT	Generative pre-trained transformer
UK	United Kingdom
DECIDE-AI	Developmental and Exploratory Clinical Investigations of DEcision support systems driven by Artificial Intelligence
GDPR	General Data Protection Regulation
EU AI Act	European Union Artificial Intelligence Act
SANRA	Scale for the Assessment of Narrative Review Articles
VANRA	Verification of Assertions in Narrative Review Articles
CONSORT-AI	Consolidated Standards of Reporting Trials–Artificial Intelligence

## Appendix A. Search Term Pairs

**Table A1:** Complete term pairs used in PubMed and Scopus. PubMed: each listed pair was joined with “AND” and entered in the default search fields. Scopus: the identical pairs were joined with “AND” and entered in the default search fields. No author-selected field tags were used. Searches were conducted from 1 June to 31 July 2026 for publications from 2015 to 31 July 2026.

No.	Search Pair (Used Separately in Each Database)
1	artificial intelligence AND surgery
2	artificial intelligence AND informed consent
3	artificial intelligence AND patient communication
4	artificial intelligence AND shared decision-making
5	artificial intelligence AND decision support
6	large language models AND surgery
7	large language models AND informed consent
8	large language models AND patient communication
9	large language models AND shared decision-making
10	large language models AND decision support
11	ChatGPT AND surgery
12	ChatGPT AND informed consent
13	ChatGPT AND patient communication
14	ChatGPT AND shared decision-making
15	ChatGPT AND decision support
